# Forging of Mg-Al-Zn Magnesium Alloys on Screw Press and Forging Hammer

**DOI:** 10.3390/ma14010032

**Published:** 2020-12-23

**Authors:** Andrzej Gontarz, Krzysztof Drozdowski, Jacek Michalczyk, Sylwia Wiewiórowska, Zbigniew Pater, Janusz Tomczak, Grzegorz Samołyk, Grzegorz Winiarski, Piotr Surdacki

**Affiliations:** 1 Faculty of Mechanical Engineering, Lublin University of Technology, 20-618 Lublin, Poland; z.pater@pollub.pl (Z.P.); j.tomczak@pollub.pl (J.T.); g.samolyk@pollub.pl (G.S.); g.winiarski@pollub.pl (G.W.); piotr.surdacki@pollub.pl (P.S.); 2 ZOP Co. Ltd. Forging Plant, 21-045 Świdnik, Poland; krzysztof_drozdowski@kuznia-zop.pl; 3 Faculty of Production Engineering and Materials Technology, Czestochowa University of Technology, 42-201 Czestochowa, Poland; michalczyk.jacek@wip.pcz.pl (J.M.); wiewiorowska.sylwia@wip.pcz.pl (S.W.)

**Keywords:** magnesium alloys, die forging, screw press, hammer, mechanical properties, grain size

## Abstract

Magnesium alloys are highly strain rate sensitive and exhibit good workability in a narrow forging temperature range. Consequently, parts made of these materials are usually forged with low-speed hydraulic presses, using specially designed tool heating systems in order to ensure near-isothermal conditions. This study investigates whether popular magnesium alloys such as Mg-Al-Zn can be forged in forging machines equipped with high-speed forming tools. Experimental upset forging tests on AZ31B, AZ61A and AZ80A specimens were conducted, using a screw press with a ram speed of 0.5 m/s and a die forging hammer with a ram speed at stroke of about 5 m/s. Test specimens were preheated to 350 °C, 410 °C and 450 °C. After the upset forging process, they were air- or water-cooled and then examined for their workability, hardness and grain size. To validate the results, a forging process for a producing handle was designed and modelled by the finite element method. Distributions of strain, temperature and fracture criterion were analysed, and energy and force parameters of the forging process were calculated. After that, experimental tests were performed on AZ31B and AZ61A specimens in order to determine mechanical properties of forged parts and examine their micro- and macrostructure. Results have demonstrated that AZ80A is not suitable for forging with either the screw press or the die forging hammer, that AZ61A can be press- and hammer-forged but to a limited extent, and that AZ31B can be subjected to forging in both forging machines analysed in the study.

## 1. Introduction

Due to their low specific weight and high relative strength, magnesium alloys are used as cast or wrought alloys in many industries such as aviation and aerospace, automotive, sports and recreation, electronics, and medical [1,2,3]. New areas of application for these alloys are also developed, e.g., magnesium alloys can be used as energy absorbers due to their good vibration damping properties [4,5]. Final properties of parts made of magnesium alloys depend on the alloy type and technological operations employed to obtain a finished product. Cast magnesium alloys are usually shaped into semi-finished products by metalworking methods, primarily extrusion forging and rolling. Circular-section bars are the most widely used as billet in the forging process. Parts with flat shapes and near-rectangular profiles can also be forged from billets such as slabs or cast preforms [6], in various as-received conditions, depending on the heat treatment type. Forgings can be subjected to precipitation and age hardening, recrystallization annealing and stress relief annealing, or they can be left thermally untreated.

Strain rate and temperature of the billet and tools are the most important parameters of the forging process. Owing to their high sensitivity to strain rate, magnesium alloys should be forged predominantly using hydraulic presses or mechanical presses with low operating speeds, under isothermal conditions. Put simply, it can be stated that the higher the strength properties of an alloy, the lower the strain rate should be applied [7,8,9,10,11]. The significance of forging temperature range was proved by results obtained, among others, by Bauser et al. [12]. The study investigated the relationship between forging temperature and the workability of AZ61, concluding that even a slight decrease in the temperature of the workpiece by 4 °C (from 212 °C to 208 °C) has a negative effect on the alloy’s workability. The forging temperature range for most wrought magnesium alloys is 290–450 °C. Due to the narrow hot-working temperature range of magnesium alloys, the tools must be preheated to 205–400 °C, depending on the alloy type. The recommended temperatures of billet and tools in the forging process for selected magnesium alloys are given in [7,11,13]. The temperature range for tools is very close to or the same as that for the billet. According to study [7], for alloys susceptible to grain growth (e.g., AZ31B, AZ61A, AZ80A) forged in several operations, it is recommended that the forging temperature in successive operations be lower by approx. 15–20 °C. Forged parts should be water-cooled in order to stop further recrystallization and growth of grains. In the case of alloys subjected to dispersion hardening, the use of this of cooling method preserves alloy additions in a supersaturated condition in the solid solution, which will lead to improved strength properties in the age-hardening operation.

A large number of existing studies in the specialist literature have examined forging processes for magnesium alloys, conducted with hydraulic presses. A great practical value of these studies is that they specify parameters that should be used for a given alloy to obtain defect-free products. Płonka et al. [14] investigated the forging process of an AZ80 connecting rod. Prior to the forging process, the billets were preheated to 415 °C for 1 h in an air furnace without protective atmosphere. The temperature of the forming tools was set equal to 300 ± 10 °C. The forging process was carried out with a hydraulic press, in one ram stroke. Considering the speed of the upper die, the strain rate was set equal to approx. 0.11 s^−1^. After the forging process, forged parts were aged at a temperature of 177 ± 5 °C for 24 h and then cooled in air. Parts obtained with this forging method had correct shape and dimensions. Compared to the billet, the forged part showed a slight increase in the strength properties, but its workability would remain unchanged. The rim is one of the parts that are made of magnesium alloys. This part is widely used in cars, airplanes and helicopters. An example of a forming process for a rim made of AZ80 alloy is given in [15,16]. In the first stage, a semi-finished product was formed from a billet (tube) by backward extrusion. In this operation the material was heated to 320–380 °C. In the second stage of the forging process, a rim bottom was formed. The third stage consisted of isothermal forging of a rim ring. The tools used in the last operation had specially designed heating systems in order to maintain the temperature of the tools close to the temperature of the workpiece during this relatively slow process. As a result, the thin rim ring did not cool down due to the contact with the tools and retained the desired plastic properties. Study [16] describes the isothermal forging process for a clamp made of AZ31, using tools with a heating system. According to the authors of the study, this part was forged in one operation. No cracks or underfill were observed; the product had a good quality. The forging process was conducted with the material preheating temperature in the range of 360–400 °C. The forging process was carried out using a hydraulic press with a ram speed of 16 mm/s. Liu and Cui [17] also investigated the isothermal forging process on a hydraulic press for producing a gear from AZ31B magnesium alloy. Heating elements were mounted in the tools. The gear was forged in two operations. The preliminary forging operation was conducted with the billet preheated to 300 °C, and the tools preheated to 275 °C. In the final forging operation, the billet had a temperature of 290 °C while the tools were heated to 265 °C. The speed of the upper tool was set equal to 0.5 mm/s. Shan et al. [18] investigated the isothermal forging process for a ribbed support made of Mg-10Gd-2Y-0.5Zn-0.3Zr. The process was carried out on a hydraulic press with a load of 50 MN and a tool speed of 1 mm/s. It was performed under isothermal conditions, with the tools and billet preheated to 407 °C. A mixture of graphite and water was used for lubrication. Forged parts were subjected to ageing at a temperature of 200 °C. The aging operation significantly improved the strength properties of the products, particularly their tensile strength. Studies [19,20] describe the forging process for a door lock made of WE43 and AZ80 magnesium alloys. A press with a ram speed of 5 mm/s was used, with the tools heated with a specially designed heating system. The billet and tools were preheated to the temperature range of 300–320 °C (AZ80) and 300–350 °C (WE43). Despite the low workability of the analysed alloys, the applied temperature and speed parameters made it possible to obtain good quality products. Studies [21,22] present a forging method for ribbed parts made of AZ31 magnesium alloy. The process was carried out in a hydraulic press with three movable rams. The tools were provided with proton heaters to heat them up before the forging process. The billet was preheated to 410 °C, and the tools to 250 °C. The obtained flat products with thin ribs were correct. It is worth emphasizing that previous attempts to conduct the forging process without preheating the tools to an appropriate temperature ended up in a failure—forged parts had cracks. Numerous forging processes for magnesium alloys are reported in the specialist literature [23,24,25,26,27,28,29,30,31,32,33,34]. They have two features in common with the previously mentioned processes: all processes were carried out at low strain rates and the tools used in the processes were heated, usually to a temperature close to that of the workpiece. In effect, the forging process could be performed with high deformations, even for hard-to-deform magnesium alloys such as AZ80 or WE43. Forging plants manufacturing parts made of magnesium alloys use hydraulic presses equipped with specially designed tool heating systems to ensure near-isothermal conditions. The limitation of this technology is its low efficiency and a high cost of tools provided with such heating systems, not to mention the fact that these factors significantly increase production costs.

In the light of reported results, it is justified to investigate forging processes for selected magnesium alloys conducted using standard high-speed forging machines, such as forging hammers and screw presses. The scientific aim of this study is to determine the relationships between forging temperature and deformation speed related to forging machines as well as between cooling method and properties (workability, hardness and structure quality) of selected magnesium alloys subjected to forging with industrial forging machines such as screw presses and forging hammers. The main practical goal of the study is to develop the technological assumptions of a hot forging process for selected magnesium alloys, conducted with screw presses and forging hammers that are available in many standard forging plants.

## 2. Materials and Methods

The study was conducted on magnesium alloys containing aluminium and zinc, i.e., AZ31B, AZ61A and AZ80A. Chemical compositions of these alloys are given in Table 1. The alloys primarily differ in the Al content, which significantly affects their workability.

AZ31B (MgAl3Zn) is the most widely used magnesium alloy and is suitable for hot working. It has the following properties: ultimate tensile strength TS ≈ 240 MPa, yield strength YS ≈ 150 MPa, elongation E ≈ 9% and hardness 50–55 HB. It is used in structures that do not carry loads, e.g., various types of hinges, flaps and handles used in the design of helicopters and airplanes.

AZ61A (MgAl6Zn) is a moderately hot-workable alloy. Its properties are similar to those of low-strength structural aluminium alloys and are as follows: TS ≈ 275 MPa, YS ≈ 165 MPa and E ≈ 7% and hardness of about 60 HB. This material is used for structures that carry low static and dynamic loads, e.g., pylons and levers in the aircraft design.

AZ80A (MgAl8Zn) is hard to deform and difficult to hot-work. Its properties are similar to those of structural 6000 series aluminium alloys and are as follows: TS ≈ 330 MPa, YS ≈ 250 MPa and E ≈ 4% and hardness approx. 72 HB.

The study was conducted using an F1736A screw press with a ram speed at stroke of about 0.5 m/s and an MPM 3150 forging hammer with an average ram speed at stroke of about 5 m/s. The research was divided into three stages.

### 2.1. Stage 1

Stage 1 of the study involved performing experimental upset forging tests for the selected magnesium alloys. The objective of this stage of the study was to determine the effect of temperature, tool speed of a given forging machine and cooling method on the workability, microstructure and hardness of forged parts. Results were used as a basis for estimating whether the selected alloys could be subjected to forging with the analysed forging machines. Cylindrical specimens with a diameter of 20 mm and a height of 30 mm were preheated to temperatures of 350 °C, 410 °C and 450 °C, and then upset forged with the hammer and screw press to the height of 10 mm. The preheating temperatures of specimens were selected based on data reported in the specialist literature [7,11,13,14,15,16,17,19,20,21,22] and preliminary investigations into the workability of magnesium alloys in the temperature range of 300–480 °C, conducted prior to the principal stage of the study described in the manuscript. The tools were preheated to approx. 300 °C using gas heaters. After upset forging, the specimens were cooled in air or in water with the temperature range of 10–40 °C. Three specimens were used in every tested variant of the forging process. Workability of the alloys was determined based on visual examination of the upset forged part surface for defects such as cracks, delamination or any other signs of lack of material cohesion. Hardness was examined by the Brinell method. Microstructural examination was performed on etched metallographic sections, by optical microscopy. Grain size was estimated by comparative method in compliance with the ASTM E112-10 standard [35], under which the relationship between the grain size number and the grain diameter is presented in Table 2. The results obtained in Stage 1 served as a basis in selecting alloys for verification in experimental tests.

### 2.2. Stage 2

**Figure 1:**
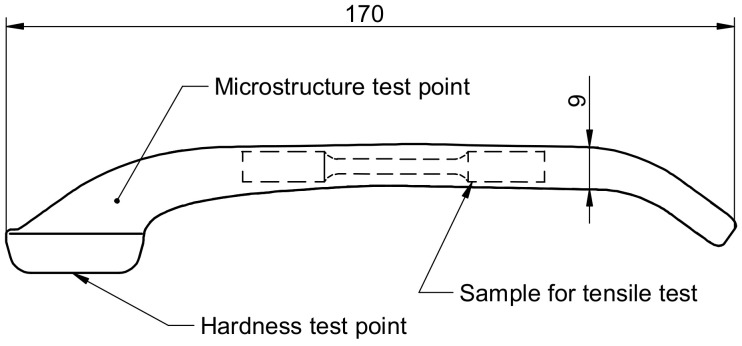
Scheme of a forged handle with location of the areas for microstructure, hardness and mechanical properties examination.

Stage 2 of the study consisted of first designing screw press and hammer forging processes and then modelling these processes to verify whether the design assumptions were correct. The study investigated a forging process for a handle shown in Figure 1. This part has a bent axis and variable section.

For the selected forging it has been assumed that it must meet the technical requirements given in Table 3.

The forging process consisted of the following:

- Cutting the billet (bar) into the dimensions of ø22 × 170 mm;
- Preheating the billet to 410 °C for 22 min (1 min per 1 mm of the billet diameter);
- Forging operation in a bending cavity;
- Preliminary forging operation in a finish forging cavity, leaving an underforged portion of about 2 mm;
- Preheating the forged part;
- Forging in a finish forging cavity;
- Cooling the forged part in air or water.

It was assumed that the dies would be heated to 300 °C.

The designed forging process was verified via numerical simulations in DEFORM 3D ver. 11.1 software based on the finite element method. The objectives of the numerical verification were the following:

- Analyse the distribution of temperature and effective strain in the forged part;
- Identify the regions of the forging that are most prone to crack formation;
- Examine the flow of material in individual operations, predominantly in terms of potential underfill, overlap and other shape defects;
- Determine stroke energy (hammer forging) and forming force (press forging) in order to select the most suitable forging machine.

Parameters applied in the numerical simulation were compliant with the designed process. The calculations were made for specimens of alloys AZ31 and AZ61, under the three-dimensional strain assumption. The workpiece and dies are respectively defined as rigid-plastic and rigid bodies. The workpiece was meshed using 100,000 tetrahedral elements. The material model of AZ31 was described by equation:

(1) $$\sigma =26+1156\times {\left(\varepsilon +0.001\right)}^{0.317}\times \exp\left(-1.349\cdot \varepsilon \right)\times {\dot{\varepsilon }}^{0.054}\times \exp\left(-0.0053\cdot T\right)$$

while the material model of AZ61 was described by equation:

(2) $$\sigma =2017.3\times \varepsilon^{0.1362}\times \exp\left(-0.7391\cdot \varepsilon \right)\times {\dot{\varepsilon }}^{0.0919}\times \exp\left(-0.00757\cdot T\right)$$

where

*σ*—flow stress, MPa;
*ε*—strain;
$\dot{\varepsilon }$—strain rate, s^−1^;
*T*—temperature, °C.

Function (1) was determined based on combined results reported in [6,36,37,38], whereas Function (2) was determined based on experimental findings reported in [39,40]. A constant friction model was applied in the analysis, with a friction factor of m = 0.25 [41]. The temperature of the tools was set equal to 300 °C; the temperature of the billet was set equal to 410 °C, and the air temperature was 40 °C. The coefficient of heat exchange between the material and tools was set equal to 11,000 W/m^2^·K, while that between the material and environment was 20 W/m^2^·K [17]. The Cockcroft–Latham (C-L) fracture criterion described by Cockroft and Latham [42] was used for the crack analysis. Implemented in appropriate software, modified model of this criterion was used, according to which the larger the danger of cracking is, the bigger constant *C* is, described by the equation [43]:

(3) $$C=\int_{0}^{\varepsilon }\frac{\sigma_{1}}{\sigma_{e}}d\varepsilon ,$$

where

*σ_1_*—maximum principal stress;
*σ_e_*—equivalent stress;
*ε*—strain;
*C*—integral value.

Fracture occurs when the integral reaches the critical value *C_limit_*, which can be expressed with the following equation:

(4) $$C_{limit}=\int_{0}^{\varepsilon_{f}}\frac{\sigma_{1}}{\sigma_{e}}d\varepsilon ,$$

where *ε_f_* is the fracture strain.

Nevertheless, it is problematic to determine the value of *C_limit_* in an unambiguous way because this value depends on factors such as the state of stress or strain paths. Consequently, the C–L criterion was only employed to determine the most fracture-prone regions of the sample; we did not, however, undertake to determine whether fracture would occur, and if so—at what moment.

In the employed simulation program, the boundary conditions relating to strains are defined by the geometry of forming tools, whereas the boundary conditions relating to loads are defined by the models of forging machines used. The screw press model was defined by a ram speed of 0.5 m/s, while the die forging hammer model was described with a drop part weight of 1200 kg (1000 kg ram weight + 200 kg die weight), blow energy of 36 kJ and efficiency of 80%.

### 2.3. Stage 3

Stage 3 of the study included the verification of the forging process under industrial conditions. A series of experimental die forging tests for producing a handle were carried out. The main goal of the tests was experimental verification of the designed die forging process based on the most important technological parameters identified in the first stage of this study (upset forging tests) and determined via FEM numerical modelling. The press and hammer forging tests were carried out using the same process parameters as in the second stage of the study. A lubricant based on grease and graphite was used in the preliminary and final forging operations.

The magnesium alloys tested in the third stage (AZ31B and AZ61A) belong to a group of materials which—due to a low content of alloy components—are not subjected to precipitation hardening (supersaturation and aging), because such heat treatment would not significantly improve their strength properties. Consequently, some forgings were only subjected to stress relief annealing and others were left as manufactured, i.e., without heat treatment, in order to determine the effect of annealing on the properties of the tested alloys. The forged parts were subjected to annealing at 350 °C for 1 h. The main aim of this treatment was to relieve stresses induced by the forging process.

The forged parts were then examined for quality. The examination involved the following:

- Determination of strength properties via static tensile testing in room temperature;
- Measurement of Brinell hardness;
- Examination of the macrostructure in the longitudinal and cross sections relative to the fibre direction;
- Metallographic examination of the microstructure in the etched condition.

The experimental results were used to investigate the possibility of forging the tested magnesium alloys with the use of high-speed forging machines, i.e., the screw press and die forging hammer.

## 3. Results and Discussion

### 3.1. Stage 1—Upset Forging Tests

Table 4, Table 5 and Table 6 show the screw press-forged and hammer-forged exemplary specimens that were preheated to different temperatures. The specimens of AZ31B preheated to 350 °C show the presence of delamination on their end face, irrespective of the forging method (Table 4). This temperature is found to be inadequate even though there are no cracks on the specimen flanks. The press-forged and hammer-forged specimens preheated to 410 °C and 450 °C are correct, without delamination or cracks. Thus, it can be claimed that both temperatures ensure adequate workability of AZ31B alloy in the forging process performed with the screw press and die forging hammer.

The results of the AZ61A specimens show that this alloy retains satisfactory workability when subjected to screw press forging, with the specimens preheated to 410 °C (Table 5). The hammer-forged specimens preheated to the same temperature show the presence of small cracks. This proves that this alloy can be hammer forged when preheated to this temperature but at lower strains. As for other preheating temperatures (350 °C and 450 °C), both the screw press-forged and hammer-forged parts show clear defects in the form of cracks. The results demonstrate that for the applied deformation speed, the preheating temperature of 350 °C is too low while the temperature of 450 °C causes brittle cracking (which is typical of hot working).

The results of the AZ80A specimens (Table 6) demonstrate that none of the applied preheating temperatures ensures that the alloy’s workability is adequate to ensure correctly forged parts using the screw press and die forging hammer. The deformation speeds obtained with these forging machines are too high for this alloy. Its strain rate sensitivity is evident when comparing the results obtained with the two forging methods; the press-forged specimens preheated to 350 °C and 410 °C have significantly fewer defects than those forged with the hammer (Table 6). It should also be noted that the specimens preheated to 350 °C have an irregular shape. The specimens preheated to 450 °C undergo brittle fracture when forged with the use of both machines.

**Figure 2:**
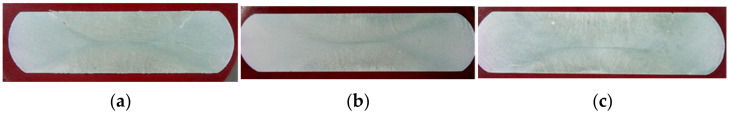
Macrostructure of selected hammer-forged specimens: (**a**) AZ31B specimen preheated to 350 °C, (**b**) AZ31B Scheme 410 °C, (**c**) AZ61A specimen preheated to 410 °C.

Macrostructure of the upset forged parts was examined for internal and subsurface defects that were impossible to identify via visual inspection. Figure 2 shows examples of the macrostructure of the specimens and defects detected by stereoscopic microscopy. It can be observed that the AZ31B specimens preheated to the lowest tested temperature of 350 °C are prone to fracture, especially at a transition point between the high strain zone and the lower strain zone (Figure 2a). The macrostructure of the upset-forged specimens of both AZ31B and AZ61A preheated to 410 °C is correct (Figure 2b,c). It should be added that some specimens have minor surface defects, predominantly on their ends (Figure 2c), which is due to the sharp edge resulting from billet cutting. It is, therefore, recommended that the billet should be chamfered before forging.

The results of the upset forging tests demonstrate that AZ31B shows relatively good workability at an appropriate preheating temperature, the workability of AZ61A is limited, while AZ80A is not suitable for press and hammer forging at all. Therefore, further part of the study focused on the first two magnesium alloys.

**Figure 3:**
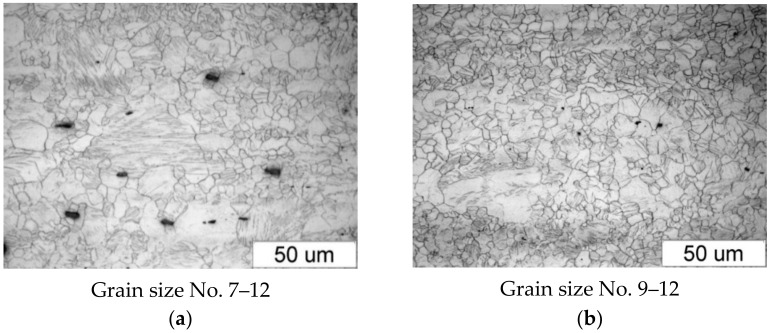
Microstructure of the billet (extruded bar) made of (**a**) AZ31B and (**b**) AZ61A.

Figure 3 shows the microstructure of the specimens (extrusion bars) before upsetting. It can be observed that the structure of both alloys is inhomogeneous, with the areas of different grain sizes. By estimating the grain size with the comparative method specified in the ASTM E112-10 standard [35], the following can be stated:

- The grain size in the AZ31B specimens corresponds to the grain size numbers 7–12;
- The grain size in the AZ61A specimens corresponds to the grain size numbers 9–12.

It must be stressed that the lower the grain size number specified in the standard, the bigger the size of the grain is (see Table 2).

**Figure 4:**
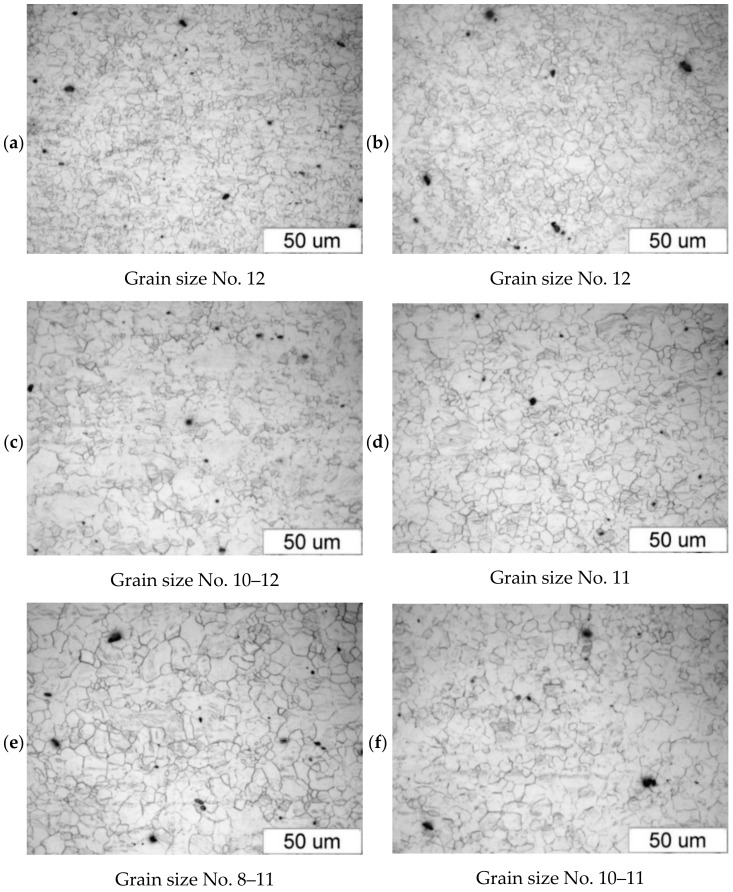
Microstructure of the AZ31B specimens after upset forging and air cooling, for different forging machines and billet preheating temperatures: (**a**) 350 °C, screw press; (**b**) 350 °C, hammer; (**c**) 410 °C, screw press; (**d**) 410 °C, hammer; (**e**) 450 °C, screw press; (**f**) 450 °C, hammer.

Figure 4 shows the microstructure of the upset forged AZ31B specimens preheated to different temperatures and forged with different speeds, subjected to air cooling. The figure also shows the grain size number according to the standard [35]. There is a clear relationship between the temperature and the microstructure of the forged parts—the grain size increases with temperature. The grain size of the specimens preheated to 350 °C corresponds to the grain size number 12 under the standard (irrespective of the forging machine applied), while that of the specimens preheated to 450 °C corresponds to the grain size numbers 8–11 (screw press) and 10–11 (hammer). The results show that the grain size obtained in the hammer forging process is slightly smaller than that obtained in press forging.

**Figure 5:**
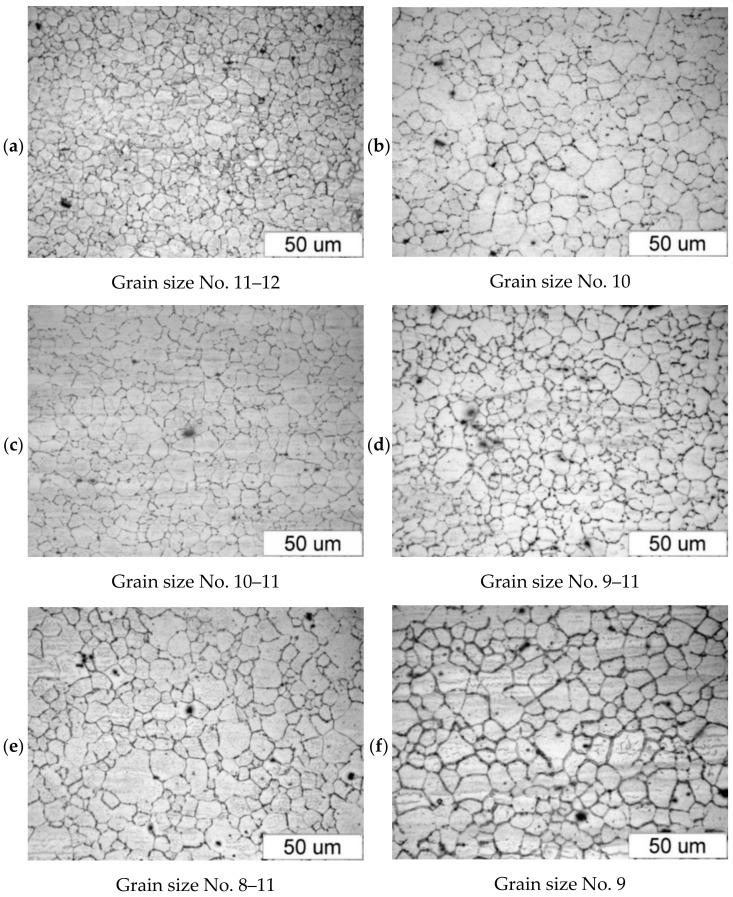
Microstructure of the AZ61A specimens after upset forging and water cooling, for different forging machines and billet preheating temperatures: (**a**) 350 °C, screw press; (**b**) 350 °C, hammer; (**c**) 410 °C, screw press; (**d**) 410 °C, hammer; (**e**) 450 °C, screw press; (**f**) 450 °C, hammer.

Figure 5 shows the microstructure of the AZ61A specimen forged with the screw press and forging hammer, with the specimens preheated to 350 °C, 410 °C and 450 °C, and then cooled in water. The relationship between the grain size and the temperature is similar to that observed for the AZ31B specimens, i.e., the grain size increases with temperature. There are no significant differences between the structure obtained in the press and the hammer forging process. An important observation is that there are traces of overheating in the structure of the hammer-forged specimens that were preheated to 450 °C, which makes it possible to conclude that this temperature is too high.

**Figure 6:**
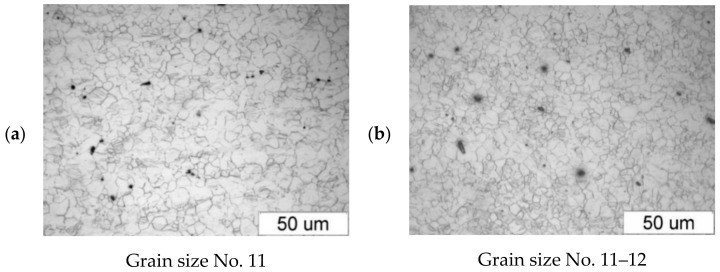

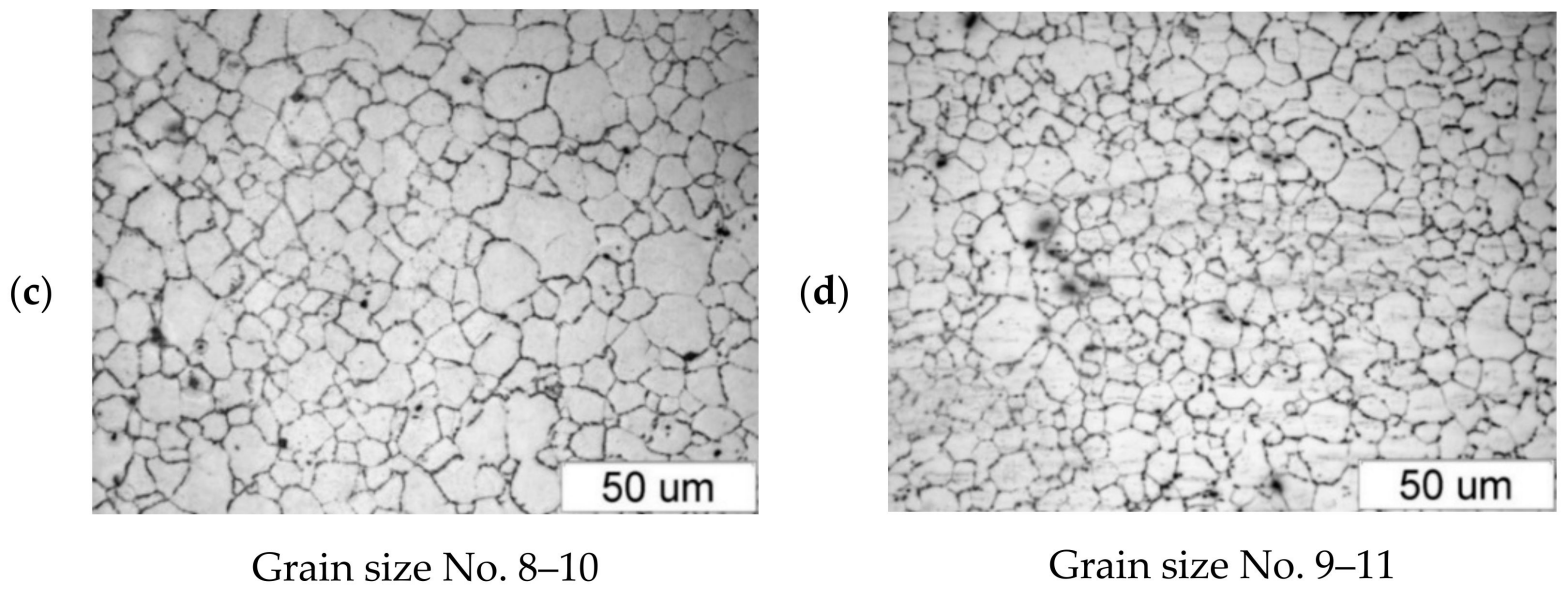
Microstructure of the hammer-forged specimens of the tested magnesium alloys, preheated to 410 °C and cooled in air and water: (**a**) AZ31B, air; (**b**) AZ31B, water; (**c**) AZ61A, air; (**d**) AZ61A, water.

Figure 6 shows the microstructure of the tested magnesium alloy specimens, depending on the cooling method after the forging process. The water-cooled forged parts have a slightly smaller grain than the air-cooled specimens. Rapid cooling reduces the grain growth. Since the difference in the structure is insignificant, it is recommended using air cooling in order to avoid unnecessary residual stresses.

Table 7 gives the hardness results of the AZ31B and AZ61A specimens. The results demonstrate that the hardness changes to a relatively small degree and practically does not depend on the temperature and cooling method. Therefore, it can be concluded that these alloys do not undergo precipitation hardening during rapid water-cooling. The cooling medium has only a minor effect on the grain size.

### 3.2. Stage 2—Numerical Modelling

**Figure 7:**
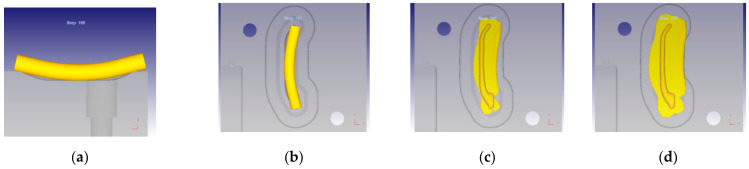
FEM modelled operations of the forging process for a handle: (**a**) bending of the billet, (**b**) bent billet is put in the die impression, (**c**) preliminary forging operation, leaving an underforged portion of approx. 2 mm, (**d**) final forging operation.

The designed forging process for producing a handle was modelled numerically. Separate simulations were carried out for the screw press forging process conducted with a pressure of 6.3 MN and for the hammer forging process conducted with a blow energy of 36 kJ. Individual operations of the forging process (bending, preliminary forging with underforged portion and final forging) are shown in Figure 7. The results were used to plot the distributions of strains, temperatures and the C–L fracture criterion. It should be noted that very similar results were obtained for the two alloys; therefore, only selected examples are presented below.

**Figure 8:**
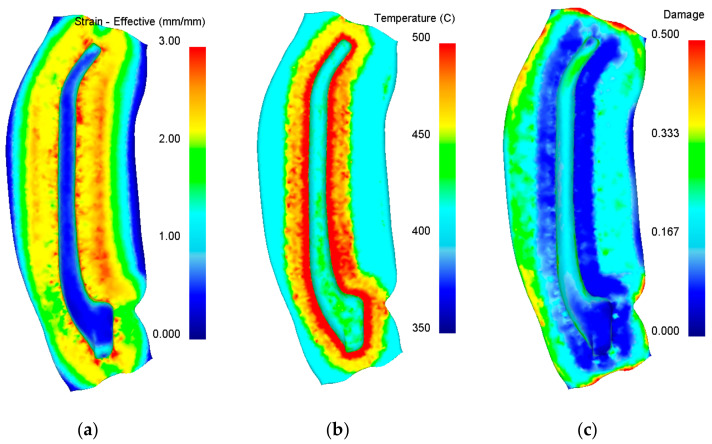
Numerical results of the hammer forging for a handle, with the distributions of: (**a**) effective strain, (**b**) temperature, (**c**) C–L ductile fracture criterion.

Distributions of effective strain during the final forging operations of the hammer forging process are plotted in Figure 8a. The distributions of this parameter are inhomogeneous. The highest strains are located at a zone between the impression and the flash, which is characteristic of die forging processes. An analysis of the forging shape shows that the die impression is completely filled and thus forging defects such as overlap should not occur. The volume of the flash is relatively large because the diameter of the billet was selected according to the biggest cross section of the detail. In terms of material yield, the forging process could be further optimized by the use of a preform with a variable cross section. However, after a cost analysis, the designed forging process was ultimately left unmodified in experimental tests.

**Figure 9:**
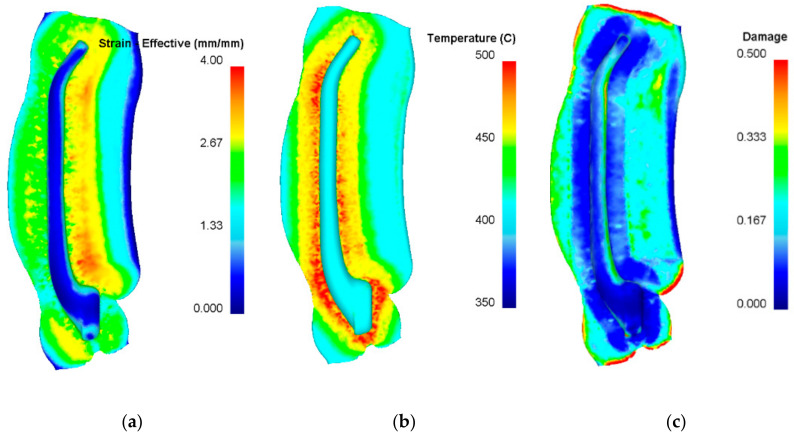
Numerical results of the screw press forging for a handle, with the distributions of (**a**) effective strain, (**b**) temperature and (**c**) C-L ductile fracture criterion.

A characteristic of the die hammer forging process is that high values of blow energy are converted into plastic deformation work which, in turn, is predominantly converted into heat. The temperature of the forging increases, especially in the area of the highest strains. Example of temperature variations toward the end of the final forging operation is plotted in Figure 8b. It should be emphasized that the temperature of the entire volume of the handle does not exceed 450 °C; therefore, it was assumed that in this part of the drop forging, material overheating should not occur. Besides their distributions of strains and temperature, the forged parts were examined for fracture by the normalized Cockcroft–Latham (C–L) criterion implemented in the DEFORM 3D ver. 11.1 software. Example of the distribution of the C–L criterion in the forged part after the final forging operation is shown in Figure 8c. The results demonstrate that the highest values of the C–L criterion are located on the flash edges. This location has practically no impact on the quality of a forged part. The numerical results of strains in the die forging process conducted with the screw press are comparable to those obtained in the hammer forging process. The temperature of the workpiece and flash is lower, which is indirectly related to the lower speed deformation. Moreover, the values of the C–L criterion are lower, which should result in a more stable forging process. Figure 9 gives the numerical results of the above parameters, obtained toward the end of the final forging operation of the press forging process.

The FEM analysis involved determination of the energy required to form a part in the preliminary and final forging operations of the hammer forging process and calculation of the maximum forming force for these operations in the press forging process. The calculations were made for AZ61A alloy which requires the use of forging machines with higher energy and load parameters than in the case of AZ31B. The results demonstrate that the preliminary forging operation of the hammer forging process was conducted with a stroke energy of approx. 20.7 kJ, while the forging operation in the finish forging cavity was conducted with a stroke energy of approx. 30.3 kJ. This means that in the hammer forging process conducted with a stroke energy of 36 kJ and an efficiency of 80%, the final forging operation must be performed at least with two strokes. The maximum force in the preliminary forging operation of the press forging process for AZ61A is approx. 1.76 MN, and it increases to approx. 2.56 MN in the final forging operation. The results of the force in the press forging process show that the screw press is suitable for conducting the designed forging process.

### 3.3. Stage 3—Experimental Tests

**Figure 10:**
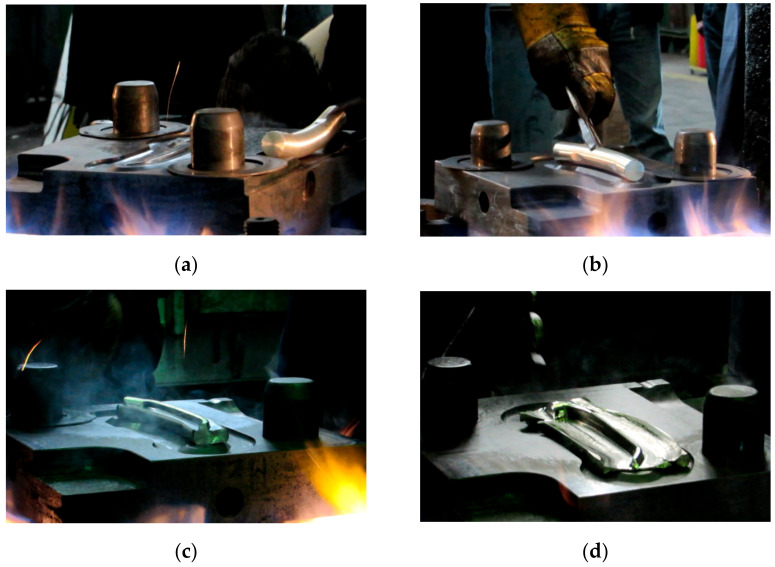
Press forging process for a handle: (**a**) bending of the billet, (**b**) bent billet is put in the die impression, (**c**) preliminary forging operation, (**d**) final forging operation.

The third stage of the study consisted of performing experimental tests of the screw press and hammer forging processes, according to the designed technique. Individual stages of the die forging process are given in Figure 10. The sequence of operations performed in the screw press and hammer forging processes was the same. The press forging process was performed with one stroke in both preliminary and final forging operations. As for the hammer forging process, the optimal solution was to deliver a greater number of strokes with a lower energy than assumed. A total of 4 to 5 strokes were delivered in the preliminary forging operation, and 2 strokes were delivered in the final forging operation. Following the recommendations given in the specialist literature, the temperature of the dies was maintained at about 300 °C during the forging process, which was ensured by the use of gas burners. Obtained forged parts were air-cooled. Some of them were left without further treatment, while others were subjected to stress relief annealing according to the time-temperature parameters specified in Section 2.

**Figure 11:**
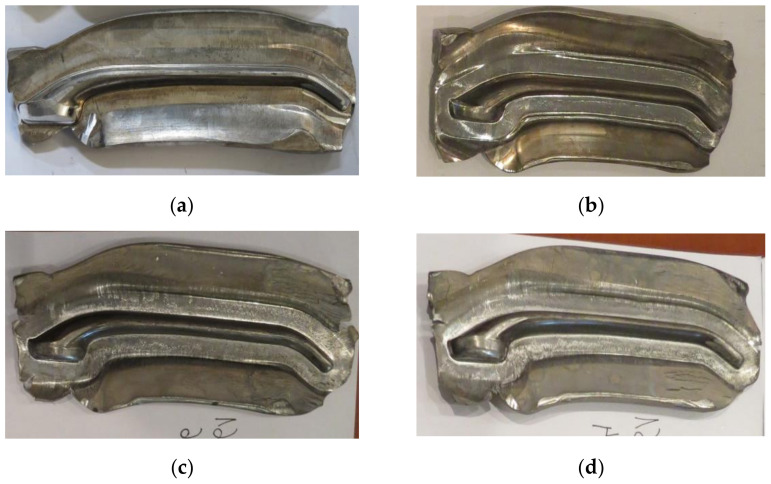
Drop forgings made in industrial conditions: (**a**) AZ31B, screw press; (**b**) AZ31B, forging hammer; (**c**) AZ61A, screw press; (**d**) AZ61A, forging hammer.

The screw press forging process is very stable. After visual inspection, the forged parts made of AZ31B and AZ61A are classified as correct—they have no defects such as cracks and delamination either in the forging or the flash (Figure 11).

The quality of the hammer-forged parts is lower, particularly in the case of the AZ61A specimens. Numerous cracks are present in the flash area. The simulation results show that the strain and, consequently, material temperature in this area are the highest, with the temperature exceeding 450 °C (Figure 8). Under such conditions brittle cracks appear in the AZ61A magnesium alloy, as shown by the upset forging tests. Regarding the hammer forging process for the two alloys, it can be observed that the application of a greater number of hammer blows with a simultaneous reduction of their energy results in a more stable behaviour of the material during the process and thus a smaller number of cracks in the flash. After flash trimming, the forged parts were subjected to further processing and laboratory examination, in order to evaluate their quality and determine if they meet the technical requirements given in Table 3. Table 8 gives the mechanical properties of selected screw press-forged and hammer-forged parts.

The results demonstrate that the stress relief annealing operation has no significant effect on the mechanical properties of the alloys under study. The only observed difference is that the workability of AZ61A has decreased from approx. 12% (press) and 14.4% (hammer) without heat treatment to 10% after annealing.

Table 9 and Table 10 show examples of the microstructure of screw press-forged and hammer-forged parts together with the estimation of their grain size. The results of microstructural examination are similar for both tested alloys. Both screw press-forged and hammer-forged parts exhibit recrystallized structure. It can also be observed that the stress relief annealing operation has caused a slight increase in the grain size.

**Figure 12:**
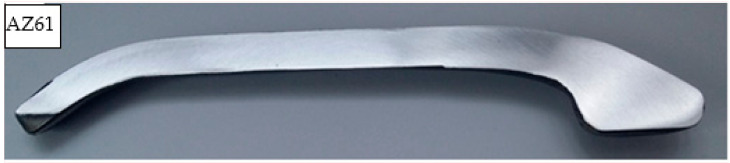
Macrostructure of a hammer-forged specimen of AZ61A.

The forged parts were also subjected to macroscopic examination for internal defects. The examination was performed on unetched metallographic sections that were cut either perpendicular or longitudinal to the fibre direction, depending on the requirement. The location of the sections subjected to macrostructural examination is shown in Figure 1. The macrostructure of the examined forged parts shows neither internal defects such as internal cracks or discontinuities, nor other surface defects such as overlap. The forged parts are also free from delamination at the die parting plane. Table 11 shows the macrostructure of the specimens of the analysed magnesium alloys after press and hammer forging. Example of the macrostructure of the specimen cut along the fibre direction is presented in Figure 12. The macrostructure of the forged parts does not show the presence of any liquation inclusions or other structural defects of this type. There are only single minor non-metallic inclusions and clusters of segregation, but their number does not exceed 4 in the entire cross-section and has an area of not more than 0.5 mm^2^ each. These defects are related to the purity of the billet and thus have no effect on the examination results whatsoever. Another surface defect to be observed is a graphite residue from the lubricant used in the forging process. Due to the presence of these surface contaminants, it is difficult to cleanse the surface of the forged parts in the etching operation. As a result, the surface of the forged parts should be first cleansed by sandblasting and then etched.

Forging tests were also made for other types of forgings. Detailed results of these studies are presented in [9,44,45].

The experimental results demonstrate that the forged parts are of good quality and have no defects such as cracks, overlap and underfill. Their mechanical properties are within the ranges specified by the standards. The tested alloys should be preheated to 410 °C, which is a temperature at which they exhibit good workability in both die hammer and press forging. It is worth mentioning that the temperature in the real forging process is slightly lower due to the heat transfer occurring when the workpiece is removed from the furnace and exposed to contact with the colder dies. The parts forged with the use of both forging machines have recrystallized microstructure.

It should be noted, however, that with increasing speed of deformation, the workability of the magnesium alloys decreases. Consequently, in terms of fracture, the hammer forging process is more difficult to conduct than the press forging process.

## 4. Conclusions

The results of this study lead to the following conclusions:

1. It is technically feasible to obtain correctly shaped forged parts with required quality from the selected AZ magnesium alloys (with zinc and aluminium addition), using high-speed tool forging machines such as screw presses and die hammers. AZ31B alloy exhibits good workability when preheated to 410 °C and forged with the use of both machines. For the same temperature, AZ61A alloy exhibits satisfactory workability when the die forging process is conducted with a screw press; when subjected to hammer forging, the workability of this alloy is acceptable only in forging processes for simple-shape parts. What is more, to obtain correctly shaped forged parts, the tools must be preheated to 300 °C.
2. Due to its low workability at high deformation speed, AZ80A alloy cannot be forged on screw presses and forging hammers, as this would lead to crack formation. AZ80A alloy parts should be forged in compliance with the guidelines presented in the specialist literature, i.e., with the use of slow hydraulic presses and tool preheating systems for ensuring near-isothermal conditions.
3. The mechanical properties of the forged parts obtained with the forging hammer and screw press are similar and meet the requirements for the tested alloys. The application of stress relief annealing does not affect their mechanical properties to any significant extent. It only slightly increases the grain size.
4. The results of microstructural examination have shown that the magnesium alloy specimens subjected to hammer forging are more fine-grained than those forged with the screw press. The grain in the water-cooled forged parts is slightly finer than in the air-cooled forgings.
5. The fact that some magnesium alloys containing zinc and aluminium can be forged using screw presses and forging hammers is of great practical importance. The results demonstrate that die forging processes for AZ31B and AZ61A can be performed in forging plants equipped with standard forging machines, and that the use of expensive tool preheating systems is not necessary. Therefore, the proposed forging technique is more cost-effective than isothermal forging with hydraulic presses.

## Figures and Tables

**Table 1:** Chemical compositions of magnesium alloys under study (wt.%).

Material	Al	Zn	Mn	Fe	Si	Cu	Ni	Mg
AZ31B	2.5–3.5	0.6–1.4	0.2–1.0	max 0.005	max 0.10	max 0.05	max 0.005	rest
AZ61A	5.8–7.2	0.4–1.5	0.15–0.5	max 0.005	max 0.10	max 0.05	max 0.005	rest
AZ80A	7.8–9.2	0.2–0.8	0.12–0.5	max 0.005	max 0.10	max 0.05	max 0.005	rest

**Table 2:** The relationship between the grain size number and the grain diameter according to ASTM E112-10 standard [35].

**Grain Size No.**	5.0	6.0	7.0	8.0	9.0	10.0	11.0	12.0	13.0
**Average Diameter, μm**	63.5	44.9	31.8	22.5	15.9	11.2	7.9	5.6	4.0

**Table 3:** Technical requirements for the properties of the handle forgings.

Mechanical Properties				
Material	Yield Strength, MPa	Ultimate Tensile Strength, MPa	Elongation, %	Brinell Hardness
AZ31B	150	240	9.0	45
AZ61A	165	275	7.0	50

**Table 4:** The forged specimens of AZ31B.

Forging Machine	350	Temperature, °C 410	450
Screw press	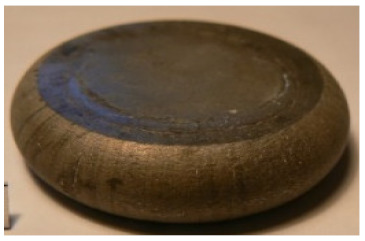	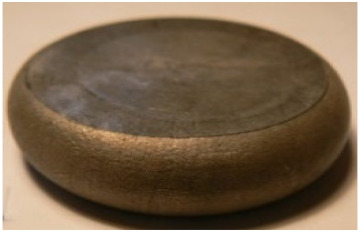	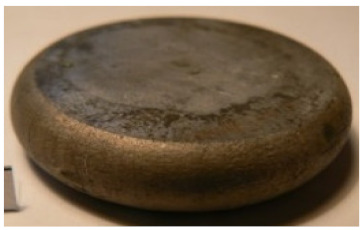
Forging hammer	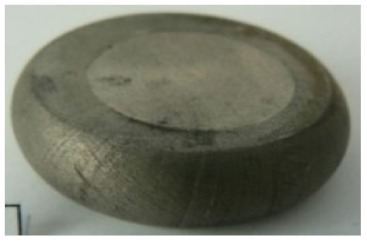	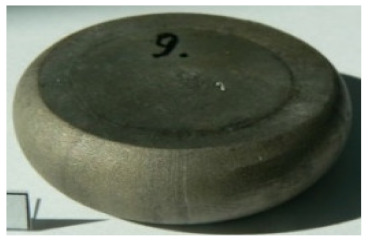	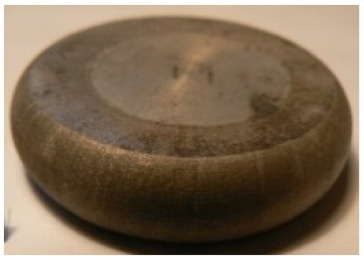

**Table 5:** The forged specimens of AZ61A.

Forging Machine	350	Temperature, °C 410	450
Screw press	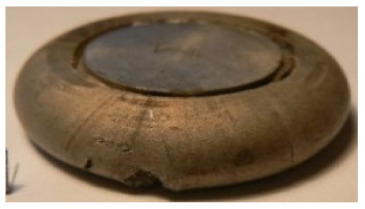	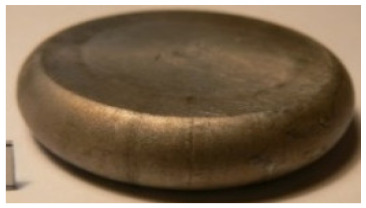	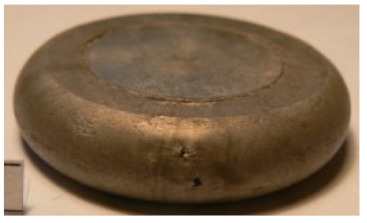
Forging hammer	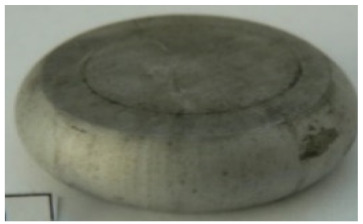	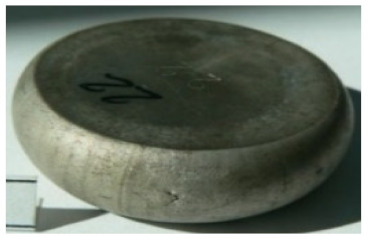	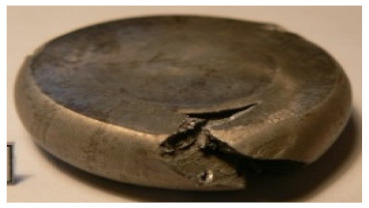

**Table 6:** The forged specimens of AZ80A.

Forging Machine	350	Temperature, °C 410	450
Screw press	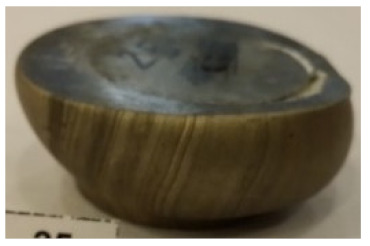	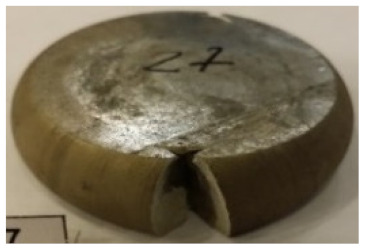	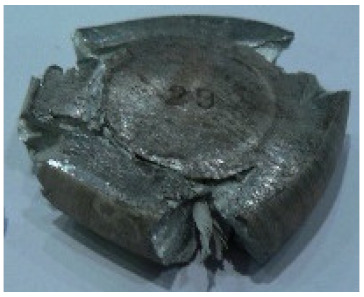
Forging hammer	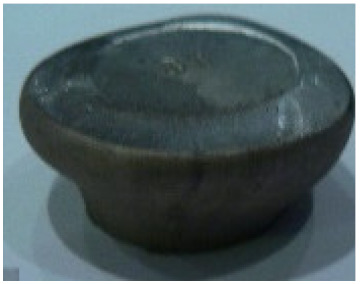	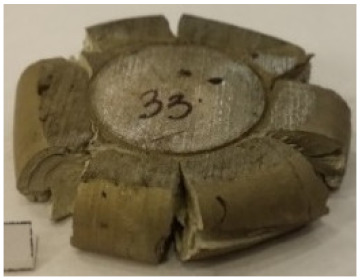	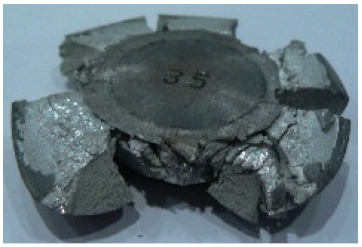

**Table 7:** Hardness of the upset forged specimens of AZ31B and AZ61A.

Temperature °C	Cooling Method	Hardness HB			
		AZ31B		AZ61A	
		Press	Hammer	Press	Hammer
350	Air	62.6	59.5	65.2	61.2
	Water	65.5	64.5	64.6	59.1
410	Air	60.5	59.3	63.2	60.0
	Water	58.9	60.4	67.6	61.9
450	Air	60.1	58.3	61.2	56.8
	Water	60.9	59.4	59.0	60.3
Billet	–	56.2		57.8	

**Table 8:** Mechanical properties of selected forged parts.

Properties	Heat Treatment	Mechanical Properties			
		AZ31B		AZ61A	
		Press	Hammer	Press	Hammer
YS, MPa	No	229	217	218	206
	SRA	218	222	220	210
TS, MPa	No	280	264	305	292
	SRA	272	271	302	292
E, %	No	14.4	14.8	12.0	14.4
	SRA	14.0	15.2	10.0	10.0
HB	No	57.2	53.2	58.3	52.3
	SRA	54.3	52.6	56.8	52.0

Description: YS—yield strength, TS—ultimate tensile strength, E—elongation, HB—Brinell hardness, SRA—stress relief annealing.

**Table 9:** Microstructure of screw press-forged parts made of AZ31B and AZ61A.

	Heat Treatment	
Alloy	No Heat Treatment	Stress Relief Annealing
AZ31B	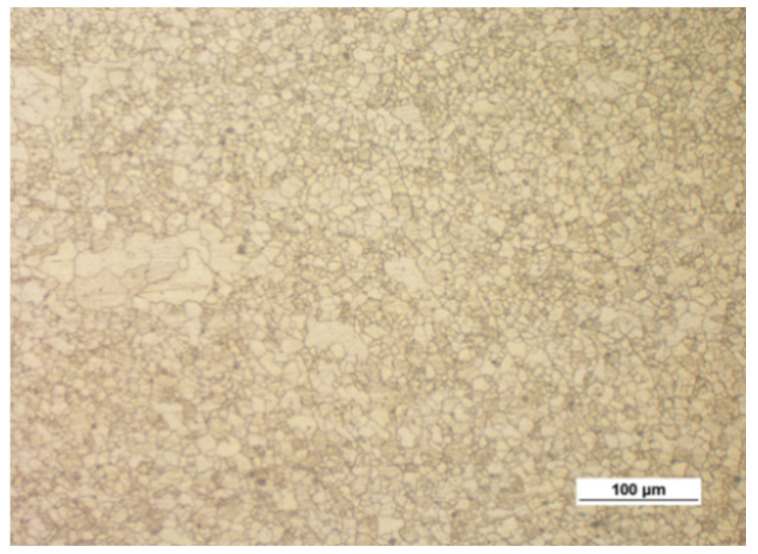 Grain size No. 9–10	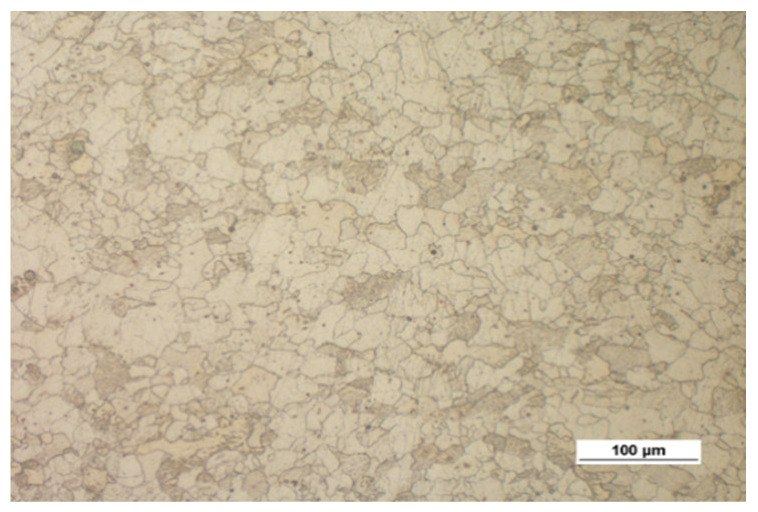 Grain size No. 8–9
AZ61A	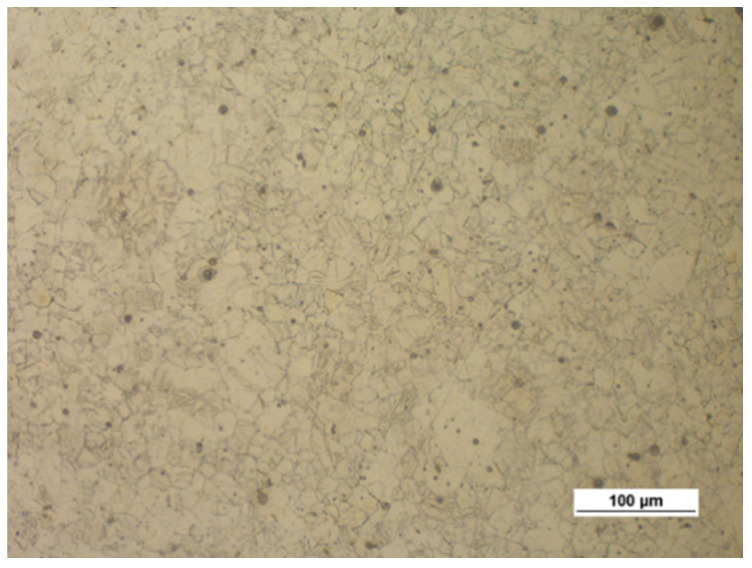 Grain size No. 8–9	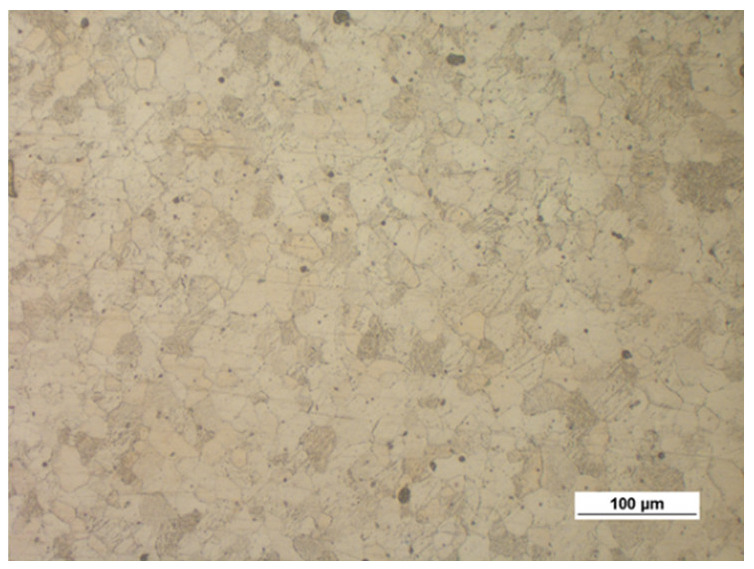 Grain size No. 7–8

**Table 10:** Microstructure of hammer-forged parts made of AZ31B and AZ61A.

	Heat Treatment	
Alloy	No Heat Treatment	Stress Relief Annealing
AZ31B	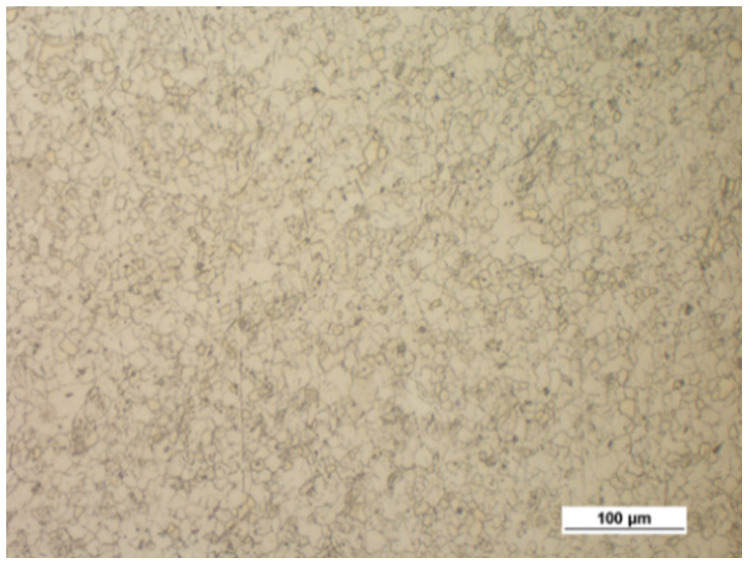 Grain size No. 8–10	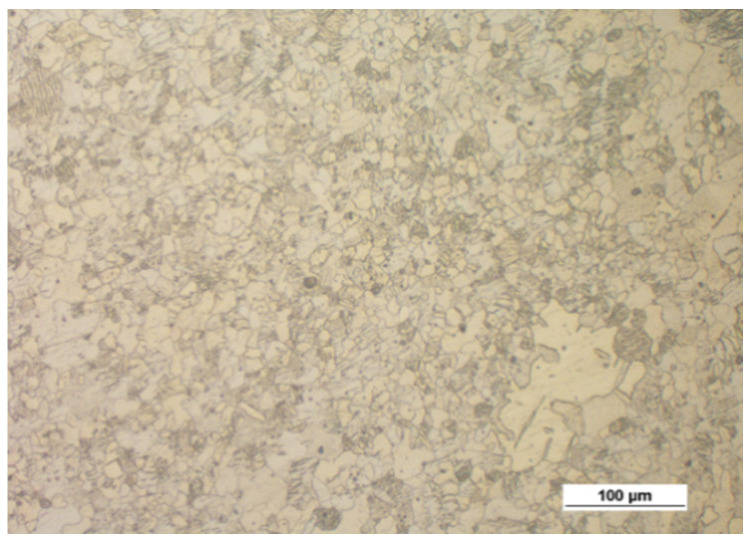 Grain size No. 7–9
AZ61A	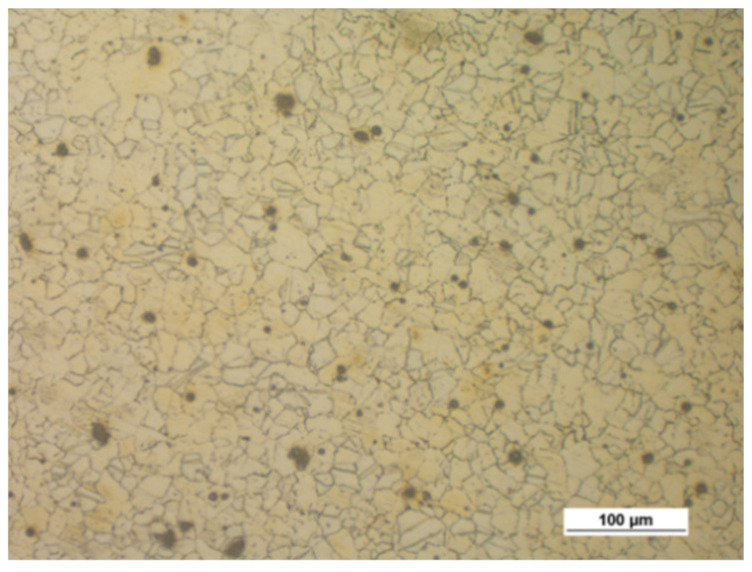 Grain size No. 8–9	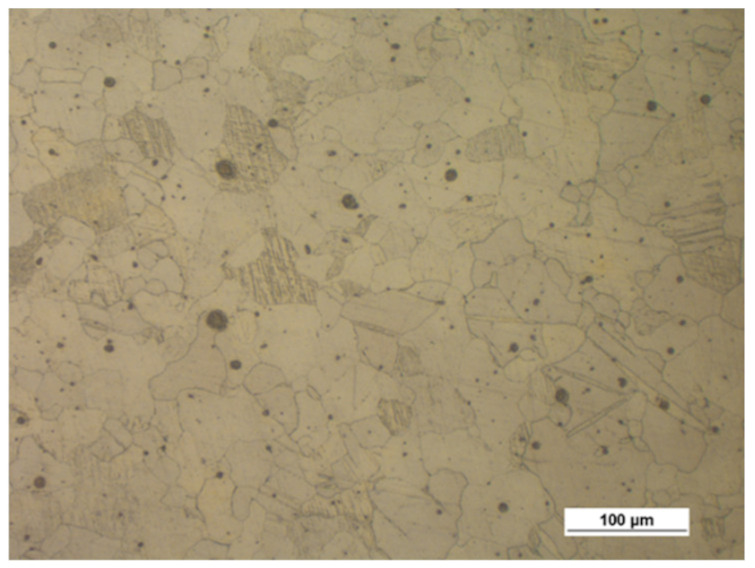 Grain size No. 6–8

**Table 11:** Macrostructure of screw-press forged and of hammer-forged specimens.

	Press	Hammer
AZ31B	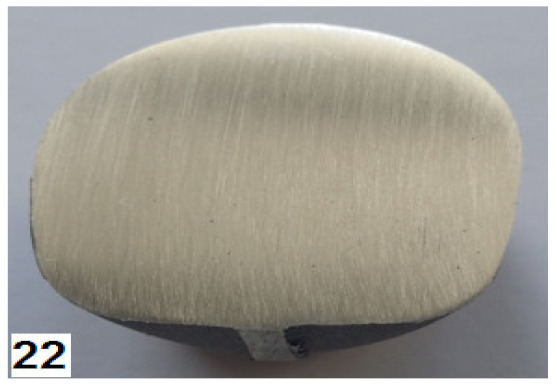	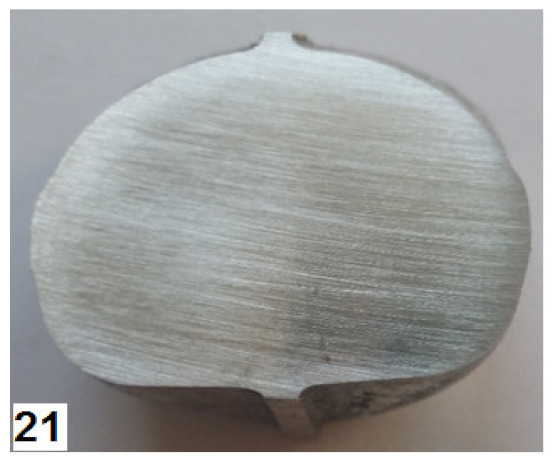
AZ61A	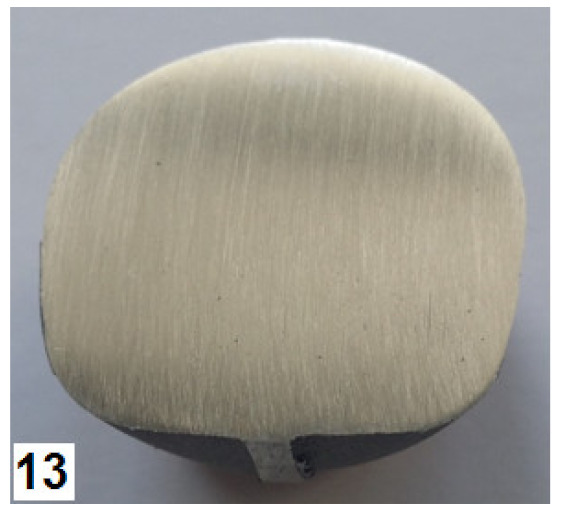	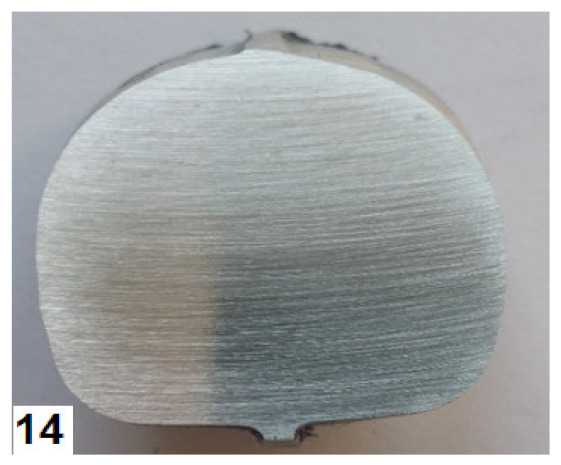

## Data Availability

Data sharing is not applicable to this article.
